# Branched-Chain Amino Acids in Parkinson’s Disease: Molecular Mechanisms and Therapeutic Potential

**DOI:** 10.3390/ijms26146992

**Published:** 2025-07-21

**Authors:** Hui-Yu Huang, Shu-Ping Tsao, Tu-Hsueh Yeh

**Affiliations:** 1 Graduate Institute of Metabolism and Obesity Sciences, College of Nutrition, Taipei Medical University, Taipei 11031, Taiwan; maggieh323@tmu.edu.tw (H.-Y.H.); d343105004@tmu.edu.tw (S.-P.T.); 2 Ph.D. Program in Drug Discovery and Development Industry, College of Pharmacy, Taipei Medical University, Taipei 11031, Taiwan; 3 Research Center for Digestive Medicine, Taipei Medical University Hospital, Taipei 11031, Taiwan; 4 Nutrition Research Center, Taipei Medical University Hospital, Taipei 11031, Taiwan; 5 Neuroscience Research Center, Taipei Medical University, Taipei 11031, Taiwan; 6 Department of Neurology, Taipei Medical University Hospital, Taipei 11031, Taiwan; 7 Department of Neurology, School of Medicine, College of Medicine, Taipei Medical University, Taipei 11031, Taiwan; 8 International Master/Ph.D. Program in Medicine, College of Medicine, Taipei Medical University, Taipei 11031, Taiwan

**Keywords:** Parkinson’s disease, branched-chain amino acids (BCAAs), branched-chain ketoacid dehydrogenase kinase (BCKDK), mitochondrial dysfunction, neuroinflammation, gut–brain axis, amino acid metabolism, precision therapeutics

## Abstract

Parkinson’s disease (PD) is a progressive neurodegenerative disorder characterized by the selective loss of dopaminergic neurons in the substantia nigra, resulting in motor symptoms such as bradykinesia, tremor, rigidity, and postural instability, as well as a wide variety of non-motor manifestations. Branched-chain amino acids (BCAAs)—leucine, isoleucine, and valine—are essential nutrients involved in neurotransmitter synthesis, energy metabolism, and cellular signaling. Emerging evidence suggests that BCAA metabolism is intricately linked to the pathophysiology of PD. Dysregulation of BCAA levels has been associated with energy metabolism, mitochondrial dysfunction, oxidative stress, neuroinflammation, and altered neurotransmission. Furthermore, the branched-chain ketoacid dehydrogenase kinase (BCKDK), a key regulator of BCAA catabolism, has been implicated in PD through its role in modulating neuronal energetics and redox homeostasis. In this review, we synthesize current molecular, genetic, microbiome, and clinical evidence on BCAA dysregulation in PD to provide an integrative perspective on the BCAA–PD axis and highlight directions for future translational research. We explored the dualistic role of BCAAs as both potential neuroprotective agents and metabolic stressors, and critically examined the therapeutic prospects and limitations of BCAA supplementation and BCKDK targeting.

## 1. Introduction

Parkinson’s disease (PD) is a progressive, multisystem neurodegenerative disorder, and the second most common after Alzheimer’s disease, affecting approximately 1% of individuals aged over 60 years and up to 4% of those beyond 80 years old [1]. As the global population continues to age, the incidence and prevalence of PD have risen dramatically, projecting to exceed 25 million by 2050 [2]. It is now recognized as the fastest-growing neurological condition worldwide in terms of prevalence, disability-adjusted life years (DALYs), and mortality [3]. These trends reflect not only increasing life expectancy, but also improvements in diagnostic sensitivity and epidemiological tracking, alongside persistent environmental exposures. Together, they place a substantial burden on patients, caregivers, healthcare systems, and the broader socio-economic landscape. Clinically, PD presents a diverse spectrum of both motor and non-motor symptoms, which vary in onset, severity, and progression. The motor hallmarks—bradykinesia, resting tremor, muscular rigidity, and postural instability—stem largely from the selective and progressive degeneration of dopaminergic neurons in the substantia nigra pars compacta. However, non-motor manifestations such as anosmia, constipation, mood disorders, cognitive decline, rapid eye movement (REM) sleep behavior disorder, and autonomic dysfunction frequently precede the onset of motor symptoms and are now recognized as integral to the disease process [1,4]. Importantly, non-motor features often emerge during the prodromal phase, highlighting the need for early biomarkers and mechanistic insights that extend beyond dopaminergic pathways. At a molecular level, PD is driven by the complex interplay of genetic susceptibility, environmental factors, and age-related cellular decline. Over 90 risk loci and genes have been identified through genome-wide association studies (GWASs) and transcriptomic analyses, collectively accounting for approximately one-third of heritable PD risk [4,5]. Pathophysiological, PD is associated with multiple cellular insults, including pathological α-synuclein aggregation (resulting in Lewy body formation), impaired mitochondrial function, chronic oxidative stress, defective autophagy and lysosomal clearance, synaptic and axonal degeneration, and persistent neuroinflammation [1]. These abnormalities interact within a complex network, ultimately leading to selective neuronal vulnerability, particularly in metabolically active brain regions such as the basal ganglia.

Recent research has pointed to amino acid metabolism—and more specifically, branched-chain amino acids (BCAAs)—as an underexplored but potentially pivotal contributor to PD pathobiology. BCAAs, consisting of leucine, isoleucine, and valine, are essential amino acids that humans must obtain from dietary sources. While traditionally associated with muscle protein synthesis and anabolic signaling, BCAAs have emerged as the key regulators of energy metabolism, mechanistic target of rapamycin complex 1 (mTORC1) activation, immune modulation, insulin sensitivity, and neurotransmitter homeostasis [6]. In the central nervous system (CNS), BCAAs serve as substrates for glutamate synthesis and can modulate excitatory and inhibitory signaling through their effects on the glutamate–glutamine cycle and gamma-aminobutyric acid (GABA) production [7].

Moreover, BCAAs cross the blood–brain barrier (BBB) via L-type amino acid transporter 1 (LAT1), which also mediates the uptake of tyrosine and tryptophan, the respective precursors for dopamine and serotonin [8]. Hence, elevated peripheral BCAA levels may competitively inhibit the entry of these aromatic amino acids, potentially altering monoaminergic neurotransmission. Such mechanisms may be especially relevant during early or prodromal PD stages, where subtle imbalances in neurotransmitter precursors may affect synaptic plasticity, behavior, or neuroprotection. The regulation of BCAA catabolism is primarily governed by the mitochondrial branched-chain α-keto acid dehydrogenase complex (BCKDH), which catalyzes the irreversible oxidative decarboxylation of BCAA-derived ketoacids. The activity of this complex is inhibited via phosphorylation by branched-chain ketoacid dehydrogenase kinase (BCKDK), and activated by dephosphorylation through protein phosphatase PPM1K (protein phosphatase, Mg^2+^/Mn^2+^ dependent 1K) [9]. When branched-chain ketoacid dehydrogenase kinase (BCKDK) is overexpressed or hyperactive (i.e., gain-of-function), it inhibits BCKDH activity, leading to the accumulation of branched-chain ketoacids (BCKAs). When present in excess, these metabolites compromise mitochondrial function, increase reactive oxygen species (ROS), cause membrane depolarization, and reduce cellular energy output, together accelerating neuronal degeneration. This condition is particularly detrimental in dopaminergic neurons, which are highly susceptible to mitochondrial oxidative stress due to their elevated metabolic demand and relatively low antioxidant defenses. While gain-of-function of BCKDK contributes to neurodegeneration, loss-of-function is also harmful: it causes systemic BCAA depletion, impairing neurotransmitter synthesis and energy homeostasis. Therefore, both BCKDK gain- and loss-of-function can be deleterious, with their specific pathogenic effects depending on cellular context and developmental stage [10,11,12].

Emerging evidence from clinical metabolomics, animal models, and microbiome studies suggests a context-dependent role of BCAAs in PD. For instance, some studies report elevated levels of isoleucine and valine in the saliva or cerebrospinal fluid of early-stage PD patients, possibly reflecting altered transport or compensatory mechanisms [13,14,15]. In contrast, advanced stages often show reduced plasma or fecal BCAAs, which correlate with disease severity and functional decline. These biphasic alterations suggest a dynamic metabolic landscape in which BCAA levels may initially rise and later become depleted due to catabolic exhaustion or gut dysbiosis [16]. Beyond intrinsic metabolic regulation, gut microbiota play a crucial role in shaping systemic BCAA availability. Several commensal taxa possess genes that synthesize or degrade BCAAs, and dysbiosis in PD is frequently characterized by reduced abundance of BCAA-producing genera such as *Prevotella* and *Faecalibacterium*, alongside an increase in pathobionts associated with neuroinflammatory signaling.

**Figure 1:**
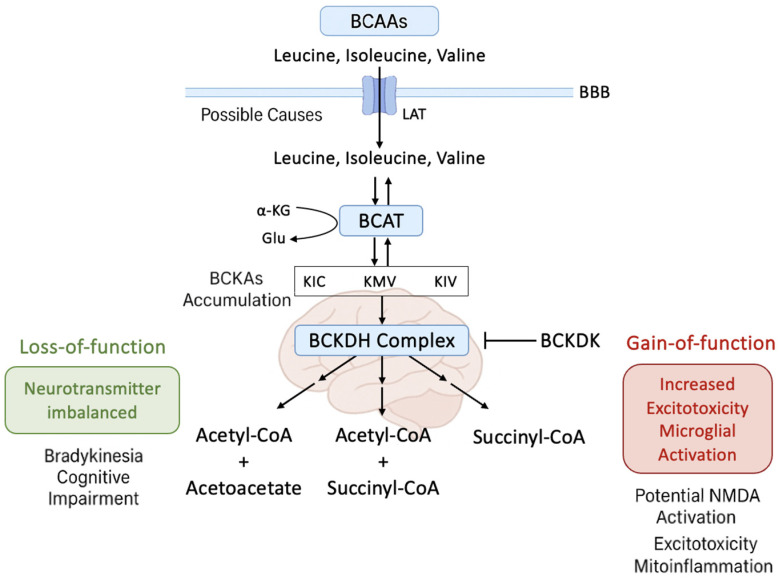
Dual neurotoxic consequences of BCKDK dysregulation in Parkinson’s disease. The schematic illustrates the bidirectional pathogenic effects of altered branched-chain α-keto acid dehydrogenase kinase (BCKDK) activity in Parkinson’s disease (PD). In the right panel (BCKDK gain-of-function), increased BCKDK activity inhibits the BCKDH complex, leading to the accumulation of branched-chain α-keto acids (BCKAs: KIC, KMV, KIV). Elevated BCKAs promote mitochondrial dysfunction, reactive oxygen species (ROS) production, glutamate excitotoxicity via N-methyl-D-aspartate (NMDA) receptor overactivation, and microglial activation, ultimately contributing to dopaminergic neurodegeneration. In the left panel (BCKDK loss-of-function), reduced BCKDK activity causes excessive branched-chain amino acid (BCAA; leucine, isoleucine, valine) catabolism and systemic BCAA depletion. This impairs neurotransmitter synthesis by disrupting the BCAT–glutamate–glutamine axis, resulting in synaptic dysfunction, bradykinesia, and cognitive decline. The figure highlights the central role of the BCKDK–BCKDH axis in regulating mitochondrial function, oxidative balance, and neurotransmitter homeostasis in PD pathophysiology.

Figure 1 presents a dual-pathogenic model of BCKDK dysregulation in PD, illustrating how both gain- and loss-of-function states lead to distinct but converging neurotoxic effects. The right panel depicts BCKDK gain of function, which inhibits BCKDH activity and causes the accumulation of branched-chain α-keto acids (BCKAs: KIC, KMV, KIV). These metabolites trigger mitochondrial dysfunction, ROS generation, NMDA receptor-mediated excitotoxicity, and microglial activation, ultimately resulting in dopaminergic neurodegeneration. In contrast, the left panel shows BCKDK loss of function, which leads to excessive BCAA catabolism and the depletion of leucine, isoleucine, and valine, disrupting neurotransmitter synthesis through the branched-chain amino acid aminotransferase (BCAT)–glutamate–glutamine cycle. This impairs synaptic transmission and contributes to motor and cognitive symptoms in PD. Collectively, the figure underscores the central role of the BCKDK–BCKDH axis in maintaining mitochondrial health, redox balance, and neurotransmitter homeostasis, highlighting its therapeutic relevance within the gut–brain metabolic interface.

Given these interconnected pathways, BCAA metabolism—and specifically the BCAA–BCKDK axis—presents a novel target for therapeutic intervention. Modulating BCAA levels, inhibiting or enhancing BCKDK activity contextually, or restoring microbial BCAA production could all represent feasible strategies to delay or modify disease progression. However, the dual role of BCAAs—as both neuroprotective agents and metabolic stressors—necessitates careful dose titration, biomarker-guided intervention, and disease-stage specificity.

This review aimed to comprehensively synthesize the existing molecular, clinical, and translational literature on the role of BCAAs in Parkinson’s disease. A special emphasis was placed on mechanistic insights into mitochondrial bioenergetics, neurotransmission, immune modulation, and gut microbiome interactions. By consolidating findings across disciplines, we aimed to identify critical knowledge gaps, clarify the temporal dynamics of BCAA alterations, and explore how targeting BCAA metabolism may open new avenues in precision neurometabolic therapy for PD.

## 2. Overview of BCAA Metabolism in Health and Disease

Branched-chain amino acids (BCAAs)—comprising leucine (LEU), isoleucine (ILE), and valine (VAL)—are among the most abundant essential amino acids in the human body, accounting for approximately 35% of the essential amino acid pool in muscle proteins. Due to the inability of mammals to endogenously synthesize these amino acids, BCAAs must be obtained entirely through dietary intake. Structurally, they are the most hydrophobic members of the amino acid family, with distinctive biophysical properties: while valine and isoleucine preferentially adopt β-sheet conformations, leucine favors α-helical arrangements [17]. These properties facilitate not only protein folding and structural stability, but also promote interactions within hydrophobic environments such as lipid bilayers and transmembrane domains, which are critical in cellular signaling and membrane transport.

Beyond their role as substrates for protein synthesis, BCAAs are increasingly recognized as key metabolic regulators that modulate a wide array of physiological processes, including energy metabolism, glucose homeostasis, gut integrity, immune surveillance, and neural communication [18,19,20]. This pleiotropic nature allows BCAAs to function as both building blocks and bioactive molecules, integrating nutritional, hormonal, and environmental cues to shape cellular responses in health and disease. BCAA catabolism is highly compartmentalized and occurs predominantly in extrahepatic tissues—particularly skeletal muscle, brain, heart, and kidney—rather than the liver, where most other amino acids are metabolized. The initial catabolic step is catalyzed by branched-chain amino acid aminotransferases (BCATs), which reversibly transfer the amino group from BCAAs to α-ketoglutarate, generating glutamate and branched-chain α-keto acids (BCKAs). Specifically, leucine yields α-ketoisocaproate (KIC), isoleucine generates α-keto-β-methylvalerate (KMV), and valine forms α-ketoisovalerate (KIV). These BCKAs are then irreversibly decarboxylated and oxidized by the mitochondrial branched-chain α-keto acid dehydrogenase complex (BCKDH), a multienzyme complex structurally analogous to pyruvate and α-ketoglutarate dehydrogenases [21]. The enzymatic activity of BCKDH is tightly regulated: it is inhibited by phosphorylation via BCKDK and activated by dephosphorylation mediated by PPM1K (also known as PP2Cm). The final products of BCAA oxidation—acetyl-CoA and succinyl-CoA—are incorporated into the tricarboxylic acid (TCA) cycle to support ATP production [22]. Compared to glucose, BCAAs offer a more efficient energy source during fasting, prolonged exercise, or catabolic stress. In parallel to their metabolic role, BCAAs serve as potent activators of the mammalian target of rapamycin complex 1 (mTORC1), a central regulator of cellular growth and anabolic processes. Leucine activation of mTORC1 leads to the phosphorylation of downstream effectors, including ribosomal protein S6 kinase (S6K) and 4E-binding protein 1 (4EBP1), ultimately enhancing mRNA translation and protein synthesis. This anabolic signaling is particularly evident in skeletal muscle, where BCAA supplementation during nutrient deprivation has been shown to enhance protein synthesis by 25–50% and reduce proteolysis by approximately 30% [23]. Furthermore, BCAAs improve glucose uptake and glycogen synthesis in both liver and muscle, linking amino acid status to insulin sensitivity and energy utilization [24].

A unique feature of BCAA metabolism is its ability to bypass hepatic first-pass clearance. Unlike other essential amino acids that are largely sequestered by the liver post-ingestion, BCAAs escape the splanchnic bed and enter systemic circulation, enabling them to exert rapid effects on peripheral tissues. However, this metabolic advantage also renders BCAA levels highly sensitive to nutritional status, physical activity, and metabolic dysfunction. Epidemiological evidence from the Women’s Health Study (*n* ≈ 19,000) revealed that elevated plasma BCAA levels are associated with higher body mass index (BMI), lower physical activity, insulin resistance, and poorer dietary quality [25]. In both children and adults, hyper-BCAAemia has been linked to obesity and type 2 diabetes mellitus (T2DM) [6,26], possibly via chronic overactivation of the mTORC1 pathway, which may impair insulin receptor signaling and exacerbate metabolic inflexibility [27]. Emerging research has also underscored the role of BCAAs in gut physiology. Leucine supports intestinal epithelial barrier integrity, regulates tight junction proteins, and promotes enterocyte proliferation, i.e., mechanisms largely mediated via mTOR activation [28,29]. However, the relationship is non-linear: moderate leucine supplementation (up to ~2.17%) exerts beneficial effects, while higher concentrations (>2.57%) appear to inhibit mucosal growth and tight junction expression, indicating a threshold beyond which therapeutic efficacy may be compromised [30].

The clinical significance of branched-chain amino acid (BCAA) dysregulation is highlighted by Maple Syrup Urine Disease (MSUD), a rare inherited metabolic disorder caused by mutations in the branched-chain α-ketoacid dehydrogenase complex (BCKDH). These mutations impair the breakdown of BCAAs, leading to the accumulation of their toxic ketoacid derivatives—KIC, KMV, and KIV—in the blood and brain [31]. If left untreated, this buildup results in encephalopathy, parkinsonian symptoms, and severe neurocognitive impairments [32]. The neurological features of MSUD emphasize the critical need for the precise regulation of BCAA metabolism to support neurotransmitter production and cerebral energy homeostasis.

Beyond monogenic disorders, aberrant BCAA profiles are increasingly implicated in a wide spectrum of chronic diseases, including cancer, cardiovascular disease, and neurodegeneration. BCAAs serve as nitrogen donors for glutamate, glutamine, and alanine synthesis, and influence key signaling cascades such as PI3K–AKT–mTOR, which orchestrate cell growth, apoptosis, and differentiation. In bone metabolism, BCAAs exhibit biphasic effects, promoting osteoclast differentiation at physiological concentrations (~400 μM) but suppressing it at supraphysiological doses (~800 μM) [33]. These findings highlight the dose and context dependency of BCAA-mediated signaling. Immunologically, BCAAs are indispensable for lymphocyte proliferation, natural killer cell function, and cytokine production. BCAA deficiency leads to immunosuppression, while supplementation has been shown to enhance immune recovery in surgical and septic patients [34,35,36]. In the nervous system, BCAAs modulate neurotransmitter availability by competing with aromatic amino acids—tryptophan (TRP) and tyrosine (TYR)—for transport across the blood–brain barrier. This competition affects brain levels of serotonin and dopamine, with potential consequences for mood, behavior, and cognition [37]. Moreover, BCAA supplementation has been associated with reduced mental and physical fatigue, especially under prolonged exertion [38].

Taken together, BCAAs are not merely nutritional components but act as integrative metabolic signals and neuromodulators. While their physiological roles in muscle anabolism, glucose metabolism, and immunity are well-established, their influence on the central nervous system—especially within the context of neurodegenerative diseases like Parkinson’s disease—remains incompletely understood. Addressing this gap may unlock novel therapeutic avenues centered on metabolic and nutritional modulation.

## 3. BCAA Alterations Across Neurodegenerative Diseases

### 3.1. Brain Uptake and Neurotransmitter Regulation

Branched-chain amino acids (BCAAs) cross the blood–brain barrier (BBB) via large neutral amino acid transporters (LAT1), which also carry tryptophan (TRP) and tyrosine (TYR)—precursors of serotonin (5-HT) and dopamine (DA), respectively [8]. This competitive transport dynamic implies that systemic BCAA elevations can reduce TRP and TYR uptake, potentially disrupting monoamine neurotransmitter synthesis and function. An acute injection of leucine, isoleucine or valine induced a decrease in brain tryptophan concentrations within 60 min, resulting in a reduction in central serotonin synthesis [39]. Although dopamine alterations are less consistently observed, studies in rodent models suggest context-dependent modulation [40,41]. Given the vulnerability of serotonergic and dopaminergic systems in Alzheimer’s disease (AD) and Parkinson’s disease (PD), even subtle shifts in amino acid competition at the BBB may influence disease progression.

### 3.2. The BCAA–Glutamate Cycle and Excitotoxicity

In the brain, BCAAs are metabolized via the BCAA–glutamine–glutamate cycle, primarily involving astrocytes and neurons [7]. Astrocytes utilize mitochondrial BCAT2 (or BCATm) to transaminate BCAAs into glutamate, which is further converted to glutamine and shuttled to neurons. There, glutaminase regenerates glutamate for synaptic release, while cytosolic BCAT1 (or BCATc) reaminates BCKAs back to BCAAs, completing the loop. This process maintains an excitatory tone and provides nitrogen for neurotransmitter turnover. However, excessive BCAA supply may increase glutamate pools beyond physiological buffering capacity, triggering excitotoxicity via the overactivation of N-methyl-D-aspartate (NMDA) receptors. This mechanism contributes to neuronal injury in AD and PD models [42,43,44]. Emerging studies also suggest an interaction with a GABAergic balance, as BCAA-derived nitrogen indirectly supports GABA synthesis, implying a broader influence on excitatory/inhibitory homeostasis.

### 3.3. Neuroinflammation and Immune Activation

BCAAs not only modulate neurotransmission, but also affect glial immune responses. Elevated BCAA concentrations activate microglia and astrocytes, promoting the release of pro-inflammatory cytokines, including IL-6, TNF-α, and IL-1β [45,46]. This low-grade neuroinflammatory state is well-documented in neurodegeneration and is thought to contribute to progressive synaptic dysfunction, oxidative stress, and blood–brain barrier breakdown. In PD, glial activation enhances neurotoxic cytokine exposure in dopaminergic-rich regions like the substantia nigra, exacerbating neuronal vulnerability.

### 3.4. BCAAs, mTOR Signaling, and Insulin Resistance

BCAAs, particularly leucine, are potent activators of the PI3K–Akt–mTOR pathway, a critical regulator of cellular growth, protein synthesis, and metabolism. Chronic BCAA-driven mTOR overactivation has been linked to insulin resistance (IR), a metabolic condition increasingly associated with AD and PD [47,48]. IR disrupts neuronal glucose uptake, reduces synaptic plasticity, and promotes tau hyperphosphorylation via GSK3β activation in AD models [49]. In PD, IR is associated with impaired mitochondrial quality control and elevated oxidative stress [50]. Thus, BCAAs may act as metabolic intermediaries that link peripheral endocrine dysfunction to central neurodegeneration.

### 3.5. Mitochondrial Stress and Redox Imbalance

Mitochondria are central hubs for both BCAA catabolism and neurodegenerative pathology. While BCAAs can support mitochondrial biogenesis and ATP production under fasting or catabolic stress, dysregulated BCAA metabolism contributes to a redox imbalance and mitochondrial injury. High intracellular BCKA levels can disrupt mitochondrial function, leading to increased reactive oxygen species (ROS) generation, mitochondrial membrane depolarization, and impaired oxidative phosphorylation. BCKA accumulation inhibits Complex I and II of the electron transport chain, which are essential for energy production and ROS control [46,51]. This disruption leads to a cascade of events, including mitochondrial damage, decreased ATP production, and increased oxidative stress. In animal studies, disrupted BCAA metabolism elevates lipid peroxidation and depletes glutathione, indicating mitochondrial oxidative damage. This phenomenon is linked to oxidative stress and ferroptosis induction in cortical neurons [52]. Dysfunctional oxidative phosphorylation exacerbates these effects by impairing energy metabolism and forcing BCAA catabolism, further contributing to oxidative stress. Altered glutamine metabolism, associated with mitochondrial dysfunction, also plays a crucial role in these pathological processes.

### 3.6. Clinical Correlations Across AD, PD, and ALS (Table 1)

Mitochondria are central hubs for both BCAA catabolism and neurodegenerative pathology. While BCAAs can support mitochondrial biogenesis and ATP production under fasting or catabolic stress, dysregulated BCAA metabolism contributes to a redox imbalance and mitochondrial injury. High intracellular BCKA levels can disrupt mitochondrial function, leading to increased reactive oxygen species (ROS) generation, mitochondrial membrane depolarization, and impaired oxidative phosphorylation. BCKA accumulation inhibits Complex I and II of the electron transport chain, which are essential for energy production and ROS control [46,51]. This disruption leads to a cascade of events, including mitochondrial damage, decreased ATP production, and increased oxidative stress.

**Table 1:** Comprehensive overview of BCAA dysregulation across neurodegenerative diseases.

Disease	BCAA Alteration	Sample/Method	Associated Mechanisms	Reference
>Alzheimer’s Disease (AD)	↑ Isoleucine, Valine	Plasma (MCI patients)	Insulin resistance, mTOR activation	[47,53]
	↑ BCAA-related genetic risk	Genetic polymorphism (isoleucine pathway)	↑ AD susceptibility	[54]
	↓ Valine	Plasma, advanced AD	Neurodegeneration, cognitive decline	[55,56]
	↓ Valine	CSF and plasma (biomarker study)	Associated with cognitive decline	[57]
	↑ Isoleucine	Plasma metabolomics	Linked to mild cognitive impairment (MCI)	[58]
Parkinson’s Disease (PD)	↑ Isoleucine, Valine	Saliva, plasma	Altered amino acid transport and LAT1 competition	[13,14]
	↓ Valine, ↑ Isoleucine (stage-specific)	CSF, brain tissue metabolomics	Mitochondrial dysfunction, α-synuclein aggregation	[15]
	↑ Ketoleucine	Metabolomic profiling	Excitotoxicity, oxidative stress	[43]
	↑ Isoleucine, Valine, Alanine, Glutamine, Histidine; ↓ Glutamate, Glucose	Serum metabolomics	Metabolic subtype differentiation (PD vs. PSP vs. MSA)	[59]
	↑ Alanine, Arginine	Plasma (correlated with L-dopa dose and disease duration)	Biomarkers of disease progression and medication load	[60]
	↑ Isoleucine, Valine	Saliva (biomarker panel)	Reflects early metabolic imbalance	[13]
	↑ Leucine (optimal ~2.14 mmol/L)	Population cohort (U-shaped risk curve)	Associated with reduced dementia risk at mid-range level	[61]
	↑ Valine, Isoleucine	Metabolomics + microbiome	Correlated with gut microbial imbalance and mitochondrial disruption	[62]
Amyotrophic Lateral Sclerosis (ALS)	↓ Valine, Isoleucine	Plasma	Metabolic exhaustion	[63,64]
	↑ Leucine, Isoleucine, Ketoleucine	CSF	Altered energy metabolism and neurotoxicity	[15]

BCAA, branched-chain amino acid; CSF, cerebrospinal fluid; MCI, mild cognitive impairment.

Clinical and translational studies reveal variable BCAA signatures across neurodegenerative diseases, likely reflecting differences in disease stage, the biofluid analyzed, and analytical technique.

In AD,

- Multiple studies report reduced valine and leucine in plasma and cerebrospinal fluid (CSF), correlating with poorer memory performance, larger ventricular volume, and faster cognitive decline [55,56,57];
- Contrasting findings show elevated BCAA levels in AD and MCI cohorts, especially in early-stage patients [47,53,58], suggesting potential stage-specific metabolic shifts;
- Genetic predisposition to higher isoleucine levels has been associated with increased AD risk [54].

In PD,

- Plasma and fecal metabolomics reveal decreased BCAAs, particularly isoleucine and valine, which inversely correlate with Hoehn and Yahr stage [65,66];
- Other studies report elevated BCAAs in early-stage PD, especially in CSF and saliva [13,15], possibly reflecting increased transport or altered peripheral metabolism.

In ALS,

- Plasma levels of valine and isoleucine are generally reduced [63], while CSF shows elevated leucine and ketoleucine [15], underscoring CNS–periphery metabolic decoupling.

These divergent findings, summarized in Table 1, highlight the need for longitudinal, biospecimen-specific studies using uniform analytic platforms.

### 3.7. Therapeutic Potentials and Risk Considerations

BCAA supplementation as a therapeutic strategy remains controversial. While moderate BCAA supplementation may enhance mitochondrial function and buffer glutamate toxicity, excessive or chronic intake may worsen excitotoxicity and oxidative damage. In ALS, high-dose BCAA supplementation was associated with increased mortality and respiratory compromise [67]. Interestingly, a study based on data from the UK Biobank suggested a non-linear relationship, with optimal mid-range leucine levels (~2.14 mmol/L) associated with the lowest risk of dementia.

### 3.8. Summary and Future Directions

BCAAs play a dual role in neurodegeneration: while essential for neurotransmission and energy homeostasis, their dysregulation may drive excitotoxicity, inflammation, insulin resistance, and mitochondrial failure. As Table 1 illustrates, BCAA patterns differ across AD, PD, and ALS in a context-dependent manner. Moving forward, multi-omic profiling, stage-specific biomarker development, and gut–brain axis exploration are the key to harnessing BCAA biology for diagnostic and therapeutic innovation in neurodegenerative diseases.

## 4. BCAAs in Parkinson’s Disease: Pathological and Mechanistic Insights

### 4.1. Central Nervous System Uptake and Dopaminergic Modulation

In Parkinson’s disease (PD), branched-chain amino acids (BCAAs) are increasingly recognized as modulators of central neurotransmitter systems. BCAAs cross the blood–brain barrier (BBB) via LAT1 transporters, which they share with tryptophan (TRP) and tyrosine (TYR), i.e., precursors of serotonin (5-HT) and dopamine (DA) [8]. Competition at LAT1 means that elevated peripheral BCAAs can restrict TRP and TYR entry into the brain, potentially altering monoaminergic signaling. While acute BCAA intake reduces brain TRP and 5-HT synthesis [39], its impact on DA is less clear. Some rodent models suggest that DA synthesis may be attenuated, whereas others report minimal changes [40,41]. Given DA’s critical role in PD pathogenesis, even subtle alterations in precursor flux may have disease-modifying implications, particularly during early or preclinical stages.

### 4.2. The Astrocyte–Neuron BCAA–Glutamate–Glutamine Shuttle in PD

Beyond precursor competition, BCAAs participate in a tightly regulated shuttle that influences synaptic glutamate availability. Astrocytic BCATs converts BCAAs into branched-chain α-keto acids (BCKAs) and glutamate, which is then converted to glutamine and transported to neurons. There, glutamine is hydrolyzed to glutamate for excitatory neurotransmission, and cytosolic BCAT1 reaminates BCKAs to regenerate BCAAs [7]. The disruption of this cycle—whether due to astrocyte dysfunction, BCKA accumulation, or aberrant BCAT activity—can tip the excitatory–inhibitory balance toward excitotoxicity, a hallmark of dopaminergic neuronal death in PD. Increased glutamate has been shown to overstimulate NMDA receptors, elevate intracellular calcium, and trigger mitochondrial permeability transitions [42,43].

### 4.3. Neuroinflammatory Activation and Cytokine Stress

Chronic neuroinflammation is a well-established driver of PD pathology. Elevated BCAA levels have been shown to activate microglia and astrocytes, increasing the secretion of TNF-α, IL-6, and IL-1β [45,46]. These pro-inflammatory cytokines propagate neuronal injury, impair autophagic flux, and exacerbate oxidative stress in PD-vulnerable regions such as the substantia nigra. Moreover, neuroinflammation and BCAA dysregulation may form a feedback loop, as mitochondrial oxidative stress can upregulate glial inflammatory responses, compounding neurodegeneration.

### 4.4. BCAAs, Insulin Resistance, and mTOR Hyperactivation

BCAAs are potent activators of the mTORC1 pathway, particularly through leucine-mediated PI3K–Akt–mTOR signaling. Sustained mTORC1 activation contributes to insulin resistance (IR), a shared comorbidity in PD and metabolic disorders. In PD models, IR has been linked to α-synuclein accumulation, mitochondrial impairment, and increased neuronal susceptibility [49,50]. In addition, impaired insulin signaling interferes with dopamine reuptake and mitochondrial autophagy (mitophagy), accelerating disease progression. Thus, BCAA-induced mTOR hyperactivation may serve as a metabolic bridge connecting peripheral endocrine dysfunction to central PD pathogenesis.

### 4.5. Mitochondrial Dysfunction, Oxidative Stress, and Dopaminergic Vulnerability

Mitochondria are central hubs for both BCAA catabolism and PD pathogenesis. While BCAAs can stimulate mitochondrial biogenesis and energy production, dysregulated BCKA accumulation impairs respiratory chain activity—particularly Complex I and II—elevating ROS and promoting mitochondrial membrane depolarization [46,51]. Dopaminergic neurons, with their high metabolic demands and low antioxidant capacity, are especially vulnerable to such stress. Moreover, interactions between BCAAs and glutamate dehydrogenase (GDH) further influence mitochondrial NADH balance and redox status, contributing to cell death under conditions of excitatory overload [44].

### 4.6. Clinical and Preclinical Evidence in PD (Table 2)

Extensive metabolomic and genetic data support a role for BCAA dysregulation in PD, as outlined in Table 2.

- Decreased BCAAs:
  (1) Zhang et al. [66] reported significantly lower plasma BCAA levels in 106 PD patients compared to 114 controls, which correlated inversely with Hoehn and Yahr stage;
  (2) Yan et al. [65] found reduced fecal BCAAs in PD patients, suggesting altered gut-derived amino acid availability;
  (3) In rotenone-induced PD mice, Yan et al. [68] observed downregulated leucine and isoleucine, linked to disease severity.
- Increased BCAAs:
  (1) Nagesh Babu et al. [59] reported elevated isoleucine and valine in PD, progressive supranuclear palsy (PSP), and multiple system atrophy (MSA);
  (2) Kumari et al. [13] found higher salivary isoleucine and valine in early-stage PD, while Figura et al. [60] observed alanine elevations in early versus advanced PD;
  (3) Wuolikainen et al. [15] detected increased CSF levels of leucine, isoleucine, alanine, and ketoleucine in PD.
- Genetic and Longitudinal Data:
  (1) Yan and Zhao [61], using Mendelian randomization in >350,000 subjects, showed genetically elevated isoleucine is associated with a lower risk of PD, supporting a potential neuroprotective window.

These findings suggest a biphasic model: early PD may be characterized by peripheral or gut-derived BCAA elevation, while advanced PD may involve central depletion or catabolic exhaustion. The heterogeneity of biospecimens (plasma, CSF, stool, saliva, urine) and analytical platforms underscores the importance of standardized, stage-stratified profiling.

### 4.7. Concluding Remarks and Future Perspectives

BCAA metabolism intersects with multiple pathogenic mechanisms in PD, including neurotransmitter dysregulation, excitotoxicity, inflammation, insulin resistance, and mitochondrial dysfunction. As demonstrated in Table 2, the directionality of BCAA alterations may depend on disease stage, sample type, and systemic metabolic state. Moving forward, precision metabolomics combined with targeted modulation of BCAA pathways may uncover novel strategies for slowing PD progression or alleviating symptom burden.

**Table 2:** Evidence from studies (animal models and clinical data) showing disease-specific changes in BCAA levels in Parkinson’s disease (PD).

Studies Subjects	Sample	Method	Findings	Reference
Decreased BCAAs				
106 PD patients and 114 controls	Plasma	HPLC-FLD	BCAAs ↓ negatively correlated with the Hoehn and Yahr stage.	[66]
20 PD patients and 20 controls	Stool	GC-MS/MS	Fecal BCAAs ↓	[65]
IEU Open GWAS project (359,194 controls and 2005 cases)	SNP	Mendelian randomization (MR) approach	Elevated levels of BCAAs, especially isoleucine, are associated with reduced risk of PD.	[61]
Rotenone-induced PD mouse model	Serum	GC-MS	Leucine and isoleucine are down-regulated and correlate with disease progression.	[68]
Increased BCAAs				
17 PD patients, 7 PSP, 6 MSA, 22 controls	Serum	NMR spectroscopy	Isoleucine ↑ valine ↑ in PD, PSP and MSA	[59]
73 PD patients; including 22 early PD (ePD), 28 advanced PD (aPD) with LID, 23 aPD without LID	Serum	HPLC-FLD	Higher levels of alanine in early PD compared with advanced PD with or without dyskinesia.	[60]
76 PD patients, 37 controls	Saliva	NMR spectroscopy	Isoleucine and valine levels are higher in PD, particularly in early-stage patients.	[13]
UK Biobank	Blood	NMR spectroscopy	Higher levels of isoleucine and leucine are associated with increased risk of PD.	[69]
20 PD patients, 20 controls	Plasma, CSF	GC-MS/MS	Plasma and CSF levels of alanine are increased in PD.	[14]
22 PD patients, 22 controls	Plasma, CSF	GC-MS/MS, LC-MS	Leucine, isoleucine, alanine and ketoleucine are increased in CSF of PD.	[15]
64 PD patients, 51 controls	Stool	GC-MS	Higher levels of leucine and isoleucine in PD fecal samples.	[62]
92 PD patients, 65 controls	Urine	GC-MS/LC-MS	Elevated levels of isoleucine and leucine in PD.	[70]
Meta-analysis	Serum		Valine, proline, ornithine and homocysteine levels were increased, while aspartate, citrulline, lysine and serine levels were significantly decreased in PD patients.	[71]
Others				
EPIC4PD cohort (total subject number: 220,494) across seven European countries. Of the 734 confirmed incident PD cases, 351 were eligible for inclusion	Serum	Untargeted metabolome by LC-MS	Three pathways were implicated in PD risk: valine, leucine, and isoleucine degradation, butanoate metabolism, and propanoate metabolism.	[72]

BCAA, branched-chain amino acid; CSF, cerebrospinal fluid; GC-MS, gas chromatography mass spectrometry; HPLC-FLD, high-performance liquid chromatography with fluorescence detection; LC-MS, liquid chromatography–mass spectrometry; LID, levodopa-induced dyskinesia; MSA, multiple-system atrophy; NMR, nuclear magnetic resonance; PD, Parkinson’s disease; PSP, progressive supranuclear palsy; SNP, single-nucleotide polymorphism.

## 5. The Gut–Brain Axis and BCAA Dysregulation in Parkinson’s Disease

### 5.1. Gut-Brain Axis Disruption as a Precursor of PD

The gut–brain axis (GBA) represents a bidirectional communication system between the gastrointestinal (GI) tract and central nervous system (CNS), integrating neural, immune, and metabolic signaling pathways. Mounting evidence suggests that GBA disruption may play a causal role in Parkinson’s disease (PD) pathogenesis [73,74]. Gastrointestinal dysfunction—manifesting as constipation, dysbiosis, and increased intestinal permeability—is not only prevalent in PD, but often precedes the onset of motor symptoms by years. This temporal relationship underpins hypotheses that the gut may act as an initiating site of disease pathology.

A dual-subtype model has been proposed: body-first PD, which begins in the gut or peripheral autonomic system and is frequently associated with REM sleep behavior disorder (RBD), and brain-first PD, which initiates in the CNS with no early peripheral signs [75]. The body-first variant implicates microbial and metabolic perturbations in disease initiation and progression.

### 5.2. Microbiota-Mediated Amino Acid Metabolism in PD

The gut microbiota exerts widespread influence on host metabolism and neurophysiology through the production of short-chain fatty acids (SCFAs), neurotransmitter precursors, and amino acids. Among these, branched-chain amino acids (BCAAs)—leucine, isoleucine, and valine—are emerging as central players in gut–brain metabolic signaling. BCAAs are synthesized by certain gut bacteria via biosynthetic genes such as ilvB, ilvC, ilvD, ilvE, brnQ, and ilvN, and are absorbed into systemic circulation, where they impact CNS neurotransmission, mTOR signaling, and mitochondrial function [76].

A study by Zhang et al. [66] reported that plasma BCAA levels inversely correlate with PD severity and are linked to microbial taxa enriched in BCAA biosynthesis genes. Notably, the depletion of key BCAA-producing bacteria such as *Coprococcus* and *Roseburia* was associated with worse motor function and reduced microbial gene expression related to amino acid synthesis. These findings underscore a direct microbiota-to-host metabolic link, wherein shifts in bacterial composition can alter systemic amino acid availability with neurophysiological consequences. This also raises the possibility that restoring microbial BCAA production capacity may represent a modifiable therapeutic target in PD.

### 5.3. Overview of Microbial Signatures in PD

Supplementary Table S1 summarizes major clinical and preclinical studies that map the association between specific microbial taxa and BCAA dysregulation in PD. A consistent pattern emerges:

- Reduced BCAA-producing taxa: Prevotella, Faecalibacterium, Roseburia;
- Increased potentially pathogenic taxa: Enterobacteriaceae, Lactobacillus, Akkermansia.

### 5.4. Barrier Dysfunction and Systemic Inflammation in PD

Gut dysbiosis compromises epithelial barrier integrity, leading to increased intestinal permeability or “leaky gut.” This facilitates the translocation of microbial components such as lipopolysaccharide (LPS) into circulation, triggering both peripheral and central immune activation [77]. Elevated LPS levels activate TLR4-mediated inflammatory cascades, leading to systemic TNF-α, IL-1β, and IL-6 elevation, which may cross the BBB and amplify neuroinflammation [78,79]. In parallel, reduced SCFA production in PD patients deprives intestinal epithelial cells and microglia of key regulatory metabolites.

### 5.5. Microbial Metabolic Networks and BCAA Compensation

Recent research has emphasized the interdependence of gut microbes in BCAA production. Cross-feeding mechanisms allow auxotrophic bacterial species to survive via the uptake of BCAAs synthesized by other microbes, maintaining community diversity and stability. Starke et al. [80] demonstrated that microbial compensation rates for leucine, isoleucine, and valine biosynthesis reach 40–41%, reflecting a shared metabolic economy within the gut ecosystem.

The disruption of these cooperative networks—via antibiotic use, diet changes, or inflammation—may collapse this mutualistic system, reducing total BCAA output and contributing to PD-related metabolic deficits. Additionally, excess lactate production by compensatory microbes may acidify the gut environment, disrupting pH-sensitive signaling and epithelial function [81].

### 5.6. Translational Relevance and Therapeutic Potential

The translational potential of targeting the microbiota–BCAA axis is gaining attention. Mendelian randomization studies indicate that genetically elevated isoleucine levels are protective against PD [82], while reduced levels are linked to disease progression [68]. Strategies that restore microbial BCAA output—such as probiotic supplementation, prebiotic fibers, and fecal microbiota transplantation (FMT)—may mitigate PD pathogenesis by reducing systemic inflammation, restoring barrier function, and enhancing mitochondrial resilience [83].

In rodent models, FMT from healthy donors improved motor symptoms, reduced α-synuclein aggregation, and normalized gut-derived amino acid profiles. Certain Lactobacillus strains, despite often being elevated in PD, have strain-specific effects: *L. reuteri*, for instance, increased BCAA availability and promoted regulatory T cell activity. These nuanced findings highlight the importance of species-level specificity when selecting microbial therapeutics. However, several challenges remain, including defining optimal timing, microbial targets, dose standardization, and ensuring long-term colonization and efficacy.

### 5.7. Conclusions and Future Directions

The gut microbiota plays a decisive role in shaping BCAA metabolism and, by extension, influences the key aspects of PD pathology, including mitochondrial function, immune activation, and neurotransmitter balance. The disruption of microbial BCAA production networks may represent an early molecular trigger for neurodegenerative cascades, particularly in body-first PD subtypes with gut-predominant pathology.

Future studies should focus on the following:

- Longitudinal profiling of BCAA-related taxa across PD stages;
- Mechanistic mapping of microbe–host metabolic interactions;
- Development of microbiome-targeted therapies that restore BCAA biosynthetic capacity while maintaining microbial ecological stability;
- Integration of gut-derived amino acid signatures as early diagnostic biomarkers, particularly in preclinical or REM sleep behavior disorder (RBD) cohorts.

Such approaches may ultimately enable precision medicine strategies that preserve metabolic integrity and slow PD progression from its earliest stages.

## 6. BCKDK and Branched-Chain Amino Acid Regulation in PD

### 6.1. BCKDK as a Key Regulator of BCAA Catabolism

Branched-chain amino acids (BCAAs)—leucine, isoleucine, and valine—are degraded via a two-step process involving first a reversible transamination to branched-chain α-keto acids (BCKAs) and second, an irreversible oxidative decarboxylation catalyzed by the branched-chain α-keto acid dehydrogenase complex (BCKDH). The BCKDH complex is composed of multiple subunits (E1α, E1β, E2, and E3), and the activity of the E1α catalytic subunit is tightly controlled via phosphorylation. BCKDK, a mitochondrial kinase, phosphorylates the E1α subunit at specific serine residues (Ser293 and Ser303), thereby inactivating the BCKDH complex. This phosphorylation is reversed by PPM1K, a mitochondrial phosphatase that dephosphorylates and reactivates BCKDH [10,21]. Through this mechanism, BCKDK functions as a metabolic gatekeeper, modulating intracellular levels of BCAAs and their corresponding BCKAs. The balance between these metabolites is crucial, as BCKAs are biologically active and can influence mitochondrial redox status, cellular ATP generation, and the biosynthesis of neurotransmitters such as glutamate and GABA. Importantly, BCKDK regulation is also responsive to cellular nutrient status and energy demand. For instance, under high-fat or nutrient-excess conditions, BCKDK is upregulated to limit BCAA oxidation and maintain intracellular pools for anabolic use. In contrast, nutrient scarcity or catabolic stress can suppress BCKDK, favoring BCAA catabolism to fuel mitochondrial respiration. This dynamic feedback places BCKDK at the core of amino acid–energy coupling across tissues.

### 6.2. BCKDK in Systemic Metabolic and Cardiovascular Health

In peripheral tissues, particularly liver, adipose, and skeletal muscle, BCKDK is intimately involved in the pathogenesis of metabolic disorders. Elevated BCKDK activity correlates with systemic accumulation of BCAAs and BCKAs, both of which are implicated in insulin resistance through mTORC1 overactivation, impaired IRS-1 signaling, and lipotoxicity. In longitudinal cohort studies, increased plasma BCAA levels predict the onset of type 2 diabetes years before clinical diagnosis.

The pharmacologic inhibition of BCKDK using BT2 has shown promising effects in murine models: it not only activates BCKDH, but also enhances whole-body BCAA oxidation, improves insulin sensitivity, reduces hepatic steatosis, and lowers circulating triglycerides [84,85]. These effects extend to skeletal muscle, where BCKDK inhibition enhances fatty acid oxidation and mitochondrial oxidative capacity.

In the cardiovascular system, BCKDK overactivity has been linked to impaired cardiac energetics. Specifically, BCAA accumulation interferes with fatty acid uptake and mitochondrial substrate flexibility. In models of cardiac hypertrophy and pressure overload, BCKDK inhibition restores cardiac contractility, reduces hypertrophic remodeling, and enhances ATP output [86]. These findings place BCKDK at the intersection of nutrient metabolism and organ-specific bioenergetics.

### 6.3. BCKDK in Cancer and Cell Signaling

In the context of cancer biology, BCKDK has recently emerged as a metabolic node that intersects with oncogenic pathways. In colorectal carcinoma, elevated BCKDK expression is not merely a metabolic adaptation but is also associated with poor differentiation, increased tumor aggressiveness, and shortened overall survival [87]. Mechanistically, BCKDK is phosphorylated at Tyr246 by Src kinase, which activates downstream MEK/ERK signaling cascades. This pathway is well known for its role in epithelial–mesenchymal transition (EMT), proliferation, and metastasis. By linking amino acid metabolism with mitogenic signaling, BCKDK acts as a dual-function molecule, facilitating both nutrient acquisition and cellular transformation. These findings suggest that BCKDK inhibition may exert anti-tumor effects via both metabolic suppression and oncogenic signal disruption, although this likely depends on tumor type and metabolic phenotype.

### 6.4. BCKDK in Neurological and Neurodegenerative Disorders

In the brain, BCKDK plays a non-redundant role in neuronal development and function. Unlike peripheral tissues where BCKDK inhibition is beneficial, in the CNS, loss of BCKDK results in severe phenotypes. BCKDK mutations in humans cause autosomal recessive neurological syndromes characterized by global developmental delay, autism spectrum disorder (ASD), epilepsy, and microcephaly [11]. These phenotypes are driven by systemic depletion of BCAAs, which impairs neurotransmitter synthesis and synaptic function.

BCAAs are precursors for glutamate and GABA—the brain’s primary excitatory and inhibitory neurotransmitters. Deficient BCAA availability impairs the synthesis of these neurotransmitters, leading to altered neuronal excitability and developmental abnormalities. Moreover, energy metabolism in neurons is highly dependent on mitochondrial health. Since BCAAs serve as anaplerotic substrates in the TCA cycle, their depletion compromises oxidative phosphorylation and redox balance, further impairing neurodevelopment.

In Parkinson’s disease, the role of BCKDK is more complex. Previous large-scale meta-analysis of genome-wide association data has suggested a potential link between the BCKDK-STX1B rs14235 genetic locus, which is involved in amino acid catabolism, and Parkinson’s disease risk [88,89]. Although one study successfully replicated the association with the rs14235A variant [12], two other studies failed to confirm this finding [90,91]. Some studies suggest that impaired BCKDK expression contributes to mitochondrial dysfunction in dopaminergic neurons, which are particularly sensitive to oxidative stress and energy failure. This aligns with the known pathology of PD, in which impaired Complex I activity and ROS accumulation play central roles. Taken together, BCKDK remains a promising candidate in PD pathogenesis due to its key role in regulating BCAA metabolism and mitochondrial function.

### 6.5. Mechanistic Insights from Experimental Models (Table 3)

Table 3 provides a detailed overview of genetic and functional studies examining BCKDK in the context of Parkinson’s disease. Several key insights emerge:

- Transcriptomic analysis in A53T-α-synuclein transgenic mice reveals significant downregulation of BCKDK in dopaminergic neurons of the substantia nigra, coinciding with onset of motor symptoms [12];
- This downregulation is associated with mitochondrial fragmentation, loss of membrane potential, elevated ROS, and reduced expression of NDUFS1, a Complex I subunit critical for NADH oxidation and ATP production;
- In vitro studies using human iPSC-derived dopaminergic neurons confirm that BCKDK knockdown sensitizes neurons to oxidative stress, increases α-synuclein aggregation, and triggers apoptotic signaling;
- Conversely, BCKDK overexpression or supplementation with BCKAs rescues mitochondrial potential and improves cell viability under toxin-induced stress conditions, such as exposure to rotenone or MPP^+^.

These findings support a model in which BCKDK serves a neuroprotective role under metabolic duress, likely by maintaining mitochondrial homeostasis and buffering intracellular BCKA flux. Notably, mitochondrial protection is coupled with reduced α-synuclein aggregation, suggesting crosstalk between metabolism and proteostasis.

**Table 3:** Summary of genetic, preclinical, and mechanistic evidence linking BCKDK to Parkinson’s disease.

Study Type	Model/Method	Key Findings and Contributions	Reference
GWAS 1	5353 PD cases vs. 5551 controls	Identified SNP rs14235 in the BCKDK–STX1B locus as associated with increased risk of Parkinson’s disease	[88]
GWAS 2 (Replication)	6476 PD cases vs. 302,042 controls (NeuroX)	Validated the association of SNP rs14235 with PD susceptibility in an independent population cohort	[88]
Epigenetic & Expression	A53T-αSyn transgenic mice, PD patient brain tissue, dopaminergic iPSC-derived neurons	BCKDK expression is significantly downregulated in PD, especially in substantia nigra dopaminergic neurons; associated with mitochondrial dysfunction via NDUFS1 and α-synuclein aggregation	[12]
Knockout Mouse Study	Bckdk^−^/^−^ and Dbt^+^/^−^ mice (deficient in BCAA metabolism enzymes)	BCKDK loss alone leads to neurodevelopmental impairment; BCAA supplementation fails to rescue phenotype, but Dbt inhibition improves behavioral and metabolic parameters	[92]

GWAS, genome-wide association studies; PD, Parkinson’s disease; BCKDK, branched-chain ketoacid dehydrogenase kinase; SNP, single-nucleotide polymorphism; αSyn, alpha-synuclein; iPSC, induced pluripotent stem cell; BCAA, branched-chain amino acid.

### 6.6. Context-Dependent Therapeutic Implications

Given its dichotomous role in peripheral and central systems, BCKDK represents a paradigmatic example of a context-dependent metabolic target. In liver and muscle, inhibiting BCKDK improves metabolic profiles and insulin responsiveness. However, in the brain, particularly in PD-affected regions, suppression of BCKDK may worsen oxidative stress, accelerate neuronal death, and disrupt neurotransmitter homeostasis.

This necessitates the development of tissue-specific pharmacological strategies:

- Selective CNS-penetrant BCKDK activators could stabilize mitochondrial function in PD;
- Peripheral-targeted inhibitors might be useful in treating diabetes and cardiovascular diseases while sparing the CNS;
- Regulatory profiling of BCKDK expression across brain regions (e.g., substantia nigra, striatum, hippocampus) is needed to understand its regional vulnerability and therapeutic windows.
- BCKDK’s interaction with mTOR, inflammatory signaling (e.g., NF-κB), and dopamine biosynthesis pathways may also uncover actionable cross-talk points for future drug development.

## 7. Targeting BCAA Metabolism in Parkinson’s Disease: Therapeutic Strategies

### 7.1. Limitations of Current Pharmacotherapy and Microbial Interference

Levodopa (L-dopa) remains the gold standard for symptomatic management of Parkinson’s disease (PD). However, its clinical effectiveness is often limited by variable patient responses and the emergence of motor complications. Recent findings highlight an underappreciated factor in this variability: gut microbial metabolism of L-dopa. Specifically, Enterococcus faecalis converts L-dopa to dopamine via a pyridoxal phosphate-dependent tyrosine decarboxylase, while Eggerthella lenta further metabolizes dopamine into m-tyramine using a molybdenum-dependent dehydroxylase [93]. These microbial enzymatic activities reduce L-dopa bioavailability, thereby diminishing therapeutic efficacy and increasing the risk of peripheral side effects. This insight opens the possibility that targeting microbial metabolism—including BCAA pathways and auxotrophic interactions—could improve drug performance by stabilizing gut-derived metabolic interference.

### 7.2. Metabolic Vulnerability in PD and the Rationale for Targeting BCAAs

PD is now recognized as a multifactorial neurodegenerative disease, where genetic predisposition, environmental exposure, and systemic metabolic dysfunction converge to drive pathology. Epidemiological studies have shown a significant association between metabolic syndrome, particularly type 2 diabetes, and increased PD risk [50,94]. Given this link, targeting energy-regulating pathways—such as branched-chain amino acid (BCAA) metabolism—has emerged as a novel therapeutic concept aimed at restoring mitochondrial function, redox balance, and neuroimmune integrity.

### 7.3. Clinical and Nutritional Trials Targeting the BCAA Axis

Although limited, clinical studies offer preliminary support for BCAA-targeted interventions. A pilot trial using whey protein supplementation—rich in both cysteine and BCAAs—resulted in reduced oxidative stress markers in PD patients but failed to yield significant clinical improvement [95]. This outcome suggests that while modulating amino acid pools may influence cellular stress responses, it may not be sufficient as monotherapy in advanced disease. Table 4 summarizes the major therapeutic approaches targeting BCAA metabolism in PD. These include the following:

- Nutritional interventions (e.g., BCAA-enriched protein supplements);
- Pharmacological modulation (e.g., BCKDK inhibitors like BT2);
- Microbiota-directed therapies (e.g., FMT, probiotic cocktails);
- Dietary restriction or rebalancing of amino acid intake.

Outcomes vary considerably based on patient age, disease stage, baseline metabolic status, and gut microbiome composition. Thus, future trials should incorporate stratification strategies that account for individual metabolic phenotypes.

### 7.4. Metabolomics-Based Evidence for BCAA Dysregulation

Extensive metabolomic profiling in PD patients has uncovered systematic perturbations in circulating metabolites, not only involving BCAAs but also other amino acids, fatty acids, bile acids, and oxidative stress markers. Notable findings include the following:

- Elevated levels of β-amino butyric acid, cystine, ornithine, phosphoethanolamine, and proline in plasma of elderly PD cohorts [96];
- Significant correlations between alanine/arginine levels and disease duration or L-dopa dosage [60];
- Upregulation of isoleucine, valine, alanine, glutamine, and histidine in serum of patients with PD, PSP, and MSA, with concurrent reductions in glutamate and glucose [59].

Paradoxically, higher blood levels of isoleucine and leucine have been linked to increased PD risk, yet are inversely correlated with dementia and Alzheimer’s disease risk [69]. These findings point to a dose- and context-dependent role for BCAAs in neurodegeneration and cognitive aging, reinforcing the importance of metabolic precision in therapeutic targeting.

### 7.5. Microbial Metabolites and BCAA Interference in the Gut–Brain Axis

BCAAs are metabolically influenced not only by host enzymes, but also by microbial contributions, particularly in the gut. Emerging work on the microbiota–brain–metabolite triad has identified three major classes of neuroactive microbial metabolites:

1. Diet-derived (e.g., SCFAs, indole derivatives);
2. Host-modified (e.g., secondary bile acids);
3. Microbe-synthesized de novo compounds (e.g., polysaccharide A).

These molecules influence immune tone, neurotransmission, epithelial barrier integrity, and neuroinflammation, often through crosstalk with cytokines (e.g., TNF-α, IL-6) and vagal afferents [79,97]. Microbial BCAA metabolism, when dysregulated, may, therefore, simultaneously impair L-dopa utilization, alter peripheral inflammation, and influence CNS amino acid pools. These pleiotropic effects reinforce the therapeutic rationale for microbiome-focused metabolic modulation in PD.

**Table 4:** Therapeutic strategies targeting the BCAA axis in Parkinson’s disease.

Strategy	Intervention Type	Mechanism of Action	Reference
Whey Protein Supplementation	Nutritional/Clinical trial	Antioxidant effect, BCAA support	[95]
BT2 (BCKDK Inhibitor)	Pharmacological	Activates BCKDH, enhances BCAA catabolism, improves mitochondrial function	[84,85]
Probiotic Therapy	Microbiota-based	Restores BCAA-producing bacteria, reduces neuroinflammation	[83]
Dietary Amino Acid Rebalancing	Nutritional	Limits aromatic amino acid competition at LAT1 transporter	[37]
Amino Acid Supplementation in Protein-Restricted PD Patients	Clinical/Dietary Intervention	Improves metabolic profile without worsening neurological symptoms; supports nitrogen balance in L-dopa users	[98]
High BCAA Diet in MPTP Mouse Model	Preclinical (MPTP-treated mice)	Modifies SCFA profile and fecal metabolites; implications for motor recovery remain unclear	[99]
Lactobacillus plantarum CCFM405	Probiotic/Gut microbiota modulation	Enhances microbial BCAA biosynthesis; modulates gut–brain axis and inflammation in PD model	[100]

BCAA, branched-chain amino acid; BCKDK, branched-chain ketoacid dehydrogenase kinase; BCKDH, branched-chain α-keto acid dehydrogenase complex; LAT1, L-type amino acid transporter 1; SCFA, short-chain fatty acid; L-dopa, Levodopa; MPTP, 1-methyl-4-phenyl-1,2,3,6-tetrahydropyridine.

### 7.6. Future Directions: Toward Precision Metabolic Therapies

The therapeutic targeting of BCAA metabolism in Parkinson’s disease represents a promising but complex frontier. Interventions must reconcile the dualistic roles of BCAAs—as both neuroprotective substrates and potential excitotoxic agents—depending on concentration, cellular context, and disease stage. Thus, designing effective strategies requires a systems-level understanding of metabolic regulation within the gut-brain axis.

Several priority areas should guide future research:

- Identification of reliable BCAA biomarkers predictive of therapeutic response;
- Integration of metabolomics and microbiome profiling to personalize treatment;
- Development of central nervous system-selective modulators (e.g., BCKDK inhibitors that spare peripheral tissues);
- Combination therapies pairing L-dopa with microbiota stabilizers to minimize microbial drug degradation.

**Figure 2:**
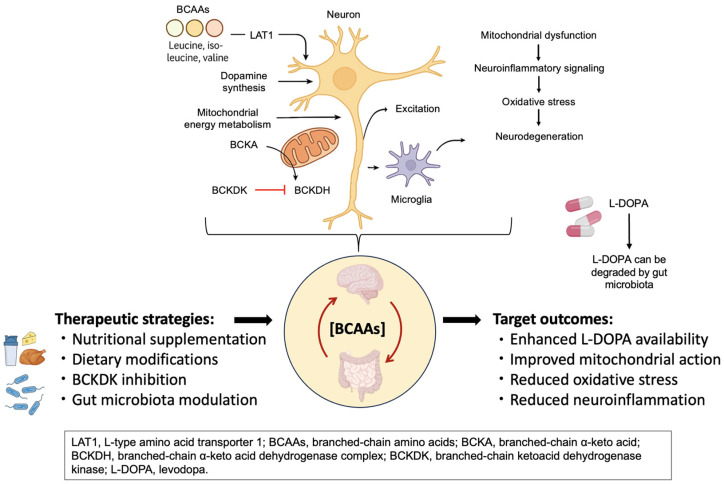
Targeting BCAA metabolism in Parkinson’s disease. This figure illustrates the key sites of BCAA-related dysregulation and therapeutic intervention along the microbiota–BCAA–brain axis in Parkinson’s disease. Strategies such as nutritional supplementation, BCKDK inhibition, and gut microbiota modulation aim to restore metabolic balance. These approaches may enhance L-DOPA availability, improve mitochondrial function, and reduce oxidative stress and neuroinflammation.

These therapeutic approaches should not only relieve symptoms, but also restore metabolic balance across the gut microbiota, systemic circulation, and central nervous system. As illustrated in Figure 2, multiple intervention points exist along the microbiota–BCAA–brain axis, including dietary modulation, microbial engineering, enzymatic regulation (e.g., BCKDK inhibition), and pharmacological strategies. Rather than being standalone metabolic tools, BCAA-targeted therapies should be viewed as integrative interventions capable of modulating neurotransmitter balance, mitochondrial function, neuroinflammation, and L-DOPA bioavailability. Realizing their full therapeutic potential will require rigorously designed clinical trials, stage-specific biomarker development, and coordinated efforts across disciplines.

## 8. Future Directions and Conclusion in BCAA Research for Parkinson’s Disease

Metabolic dysregulation is increasingly recognized as a core contributor to the pathogenesis of Parkinson’s disease (PD). Among these disturbances, reductions in circulating branched-chain amino acids (BCAAs) have drawn particular attention. Evidence suggests that diminished BCAA levels may impair mitochondrial function, elevate the production of reactive oxygen species (ROS), and exacerbate oxidative stress, thereby accelerating neurodegeneration [101,102]. Despite growing support for the pathological relevance of BCAA metabolism in PD, translating these findings into effective clinical applications remains a significant challenge.

Therapeutic strategies aimed at restoring BCAA homeostasis—such as targeted amino acid supplementation or microbiota-based interventions (e.g., probiotics or fecal microbiota transplantation, FMT)—hold considerable potential to modulate disease progression. However, these approaches must be pursued with caution. Excessive or poorly timed BCAA supplementation may trigger adverse effects such as excitotoxicity or impose additional metabolic burden on vulnerable neurons. As such, future research must address the critical parameters of dosage, treatment window, and patient-specific metabolic context to optimize safety and efficacy.

In parallel, a growing body of evidence implicates gut microbiota dysbiosis and altered microbial metabolites, including BCAAs, as contributors to PD onset and progression. Among regulatory enzymes, BCKDK (branched-chain α-ketoacid dehydrogenase kinase) has emerged as a pivotal node, linking BCAA catabolism to mitochondrial integrity and energy homeostasis. Dysregulation of BCKDK may play a direct role in PD pathogenesis, particularly by amplifying mitochondrial stress and impairing neuronal resilience.

To advance this field, future investigations should prioritize elucidating the molecular mechanisms that govern microbe–host interactions in the context of BCAA metabolism. Particular attention should be given to how microbial composition and enzymatic pathways modulate systemic and brain-specific BCAA availability, and how these interactions influence neuronal health. Identifying mechanistic targets within this axis may enable the development of personalized, metabolism-based therapeutic interventions for PD and other neurodegenerative conditions.

Emerging evidence underscores the central role of branched-chain amino acids (BCAAs) in both energy metabolism and brain function. Disruptions in BCAA homeostasis—arising from gut microbiota dysbiosis and dysregulated expression of key metabolic enzymes such as BCKDK—have been increasingly linked to the pathogenesis of Parkinson’s disease (PD). These metabolic disturbances contribute to mitochondrial dysfunction, neurotransmitter imbalance, and broader systemic metabolic stress observed in PD.

Collectively, the current findings highlight both the therapeutic promise and the complexity of targeting BCAA pathways in PD. Strategies involving BCAA supplementation or modulation of the gut microbiome hold potential to influence disease progression. However, their clinical application must account for context-dependent responses, potential risks such as excitotoxicity, and patient-specific metabolic profiles. A deeper mechanistic understanding of BCAA regulation in PD will be essential to developing safe, effective, and personalized therapeutic interventions.

## Supplementary Materials

The following supporting information can be downloaded at: https://www.mdpi.com/article/10.3390/ijms26146992/s1. References [103,104,105,106,107,108,109,110,111,112,113,114,115,116,117,118,119,120,121,122,123,124] are cited in the supplementary materials.

## Abbreviations

The following abbreviations are used in this manuscript:

4EBP1	4E-binding protein 1
5-HT	Serotonin
AD	Alzheimer’s disease
ASD	Autism spectrum disorder
BBB	Blood–brain barrier
BCAAs	Branched-chain amino acids
BCAT	Branched-chain amino acid aminotransferase
BCKAs	Branched-chain α-keto acids
BCKDH	Branched-chain α-keto acid dehydrogenase complex
BCKDK	Branched-chain ketoacid dehydrogenase kinase
BMI	Body mass index
CNS	Central nervous system
CSF	Cerebrospinal fluid
DA	Dopamine
DALYs	Disability-adjusted life years
EMT	Epithelial–mesenchymal transition
FMT	Fecal microbiota transplantation
GABA	Gamma-aminobutyric acid
GBA	Gut–brain axis
GC-MS	Gas chromatography mass spectrometry
GI	Gastrointestinal
GWAS	Genome-wide association studies
HPLC-FLD	High-performance liquid chromatography
ILE	Isoleucine
KIC	α-ketoisocaproate
KIV	α-ketoisovalerate
KMV	α-keto-β-methylvalerate
L-dopa	Levodopa
LAT1	L-type amino acid transporter 1
LC-MS	Liquid chromatography-mass spectrometry
LEU	Leucine
LID	Levodopa-induced dyskinesia
LPS	Lipopolysaccharide
MCI	Mild cognitive impairment
MPTP	1-methyl-4-phenyl-1,2,3,6-tetrahydropyridine
MSA	Multiple system atrophy
MSUD	Maple Syrup Urine Disease
NMDA	N-methyl-D-aspartate
PD	Parkinson’s disease
PPM1K	Protein phosphatase, Mg^2+^/Mn^2+^ dependent 1K
PSP	Progressive supranuclear palsy
RBD	REM sleep behavior disorder
REM	Rapid eye movement
ROS	Reactive oxygen species
S6K	S6 kinase
SCFAs	Short-chain fatty acids
SNP	Single-nucleotide polymorphism
T2DM	Type 2 diabetes mellitus
TCA	Tricarboxylic acid
TRP	Tryptophan
TYR	Tyrosine
VAL	Valine
mTORC1	Mechanistic target of rapamycin complex 1
