# Supplementary material for: Branched-Chain Amino Acids in Parkinson’s Disease: Molecular Mechanisms and Therapeutic Potential
Source: Int J Mol Sci. 2025 Jul 21;26(14):6992. doi: 10.3390/ijms26146992 (PMC12295359; doi:10.3390/ijms26146992)
Supplement: Supplementary file 1 [file ijms-26-06992-s001.zip › ijms-3728258-supplementary.pdf]

Supplementary Table S1.

Summary of Clinical and Preclinical Studies Highlighting the Role of Gut Microbiota in Regulating Branched-Chain Amino Acid (BCAA) Metabolism in Parkinson's Disease.

The table compiles key bacterial taxa identified across major PD-related microbiome studies and their associations with BCAA biosynthesis, degradation, and dysbiosis. Reduced abundance of BCAA-producing genera (e.g., *Prevotella*, *Faecalibacterium*, *Roseburia*) and enrichment of potentially pathogenic or pro-inflammatory taxa (e.g., *Enterobacteriaceae*, *Lactobacillus*, *Akkermansia*) are consistently observed across diverse populations. These microbial patterns are functionally linked to altered amino acid metabolism, mitochondrial stress, and neuroinflammatory activation.

Studies	Decreased abundance	Increased abundance	Clinical significance	References
72 PD patients, 72 controls	Prevotellaceae	Lactobacillaceae, Verrucomicrobiaceae, Bradyrhizobiaceae, Clostridiales Incertae Sedis IV, Ruminococcaceae	The prevalence of Enterobacteriaceae demonstrated a positive correlation with the severity of postural instability and gait difficulty.	[1] [2] [3]
34 PD patients, 34 controls	Bacteroidetes, Prevotellaceae	Enterobacteriaceae	The association between PD and the abundance of certain gut microbiota with reduction in fecal SCFA concentrations, which might induce alterations in the ENS and contribute to gastrointestinal dysmotility in PD.	[4] [2]
89 PD	Dorea,	Christensenella,	Gut microbiota in	[5]

patients, 66 controls	Bacteroides, Prevotella, Faecalibacterium, Stoquefichus massiliensis, Blautia glucerasea, Coprococcus eutactus, and Ruminococcus callidus	Catabacter, Lactobacillus, Oscillospira, Bifidobacterium, Ruminococcus bromii, and Papillibacter cinnamivorans	PD patients is characterized by a decrease in taxonomic diversity and significant differences in representation of 9 geneta and 15 species of microorganisms.	[2]
197 PD patients, 130 controls	Lachnospiraceae, Pasteurellaceae, Verrucomicrobiaceae	Bifidobacteriaceae, Lactobacillaceae, Tissierellaceae, Christensenellaceae	Microbiome variation may be related to the metabolism of botanical compounds and the degradation of xenobiotics.	[6] [2]
74 PD patients, 75 controls	Prevotella		The lower abundance of Prevotella bacteria in PD patients with irritable bowel syndrome.	[7] [2]
31 PD patients, 28 controls	Prevotella copri, Clostridium saccharolyticum, Eubacterium biforme	Alistipes shahii, Akkermansia Muciniphila	Differences in microbiota metabolism in PD involving the ß-glucuronate and tryptophan metabolism. The intake of either a MAO inhibitor, amantadine, or a	[8] [2] [3]

			dopamine agonist had no overall influence on taxa abundance or microbial functions.	
24 PD patients, 14 controls	genus decreased: Blautia, Faecalibacterium, Ruminococcus	Family increased: Enterobacteriaceae, Veillonellaceae, Erysipelotrichaceae, Coriobacteriaceae, Streptococcaceae, Moraxellaceae, Enterococcaceae; genus increased: Acidaminococcus, Acinetobacter, Enterococcus, Escherichia-Shigella, Megamonas, Megasphaera, Proteus, Streptococcus;	The disease severity and PD duration are negatively correlated with the putative cellulose degraders, and positively correlated with the putative pathobionts.	[9] [3]
76 PD patients, 21 iRBD, 78 controls	Melainabacteria	Verrucomicrobia, Verrucomicrobiales, Verrucomicrobiaceae, Akkermansia, Prevotella	Eighty percent of the distinct gut microbes in PD compared with iRBD were identified. In PD: Motor symptoms related to Anaerotruncus spp., Clostridium XIVa, and Lachnospiraceae,	[10] [2] [3]

			Non-motor symptoms related to Anaerotruncus, Akkermansia, and several unclassified Bacteria.	
45 PD patient, 45 controls	Lactobacillus, Sediminibacterium	Genera Clostridium IV, Sphingomonas, Aquabacterium, Clostridium XVIII, Holdemania, Anaerotruncus, Butyricicoccus	The presence of Escherichia/Shigella showed a negative correlation with the duration of disease, while Dorea and Phascolarctobacterium demonstrated a negative association with levodopa equivalent doses. Additionally, Butyricicoccus and Clostridium XlVb were found to be linked with cognitive impairment.	[11] [2] [3]
64 PD patients, 64 controls, twice on average	Family decreased: Prevotellaceae; genus decreased: Roseburia	genus increased: Bifidobacterium;	The previously detected gut microbiota differences between Parkinson's	[12] [3]

2·25 years apart			patients and controls persisted after 2 years. There was some support for a different distribution of enterotypes and a decreased abundance of Prevotella in faster-progressing patients.	
193 PD patient, 22 PSP, 22 MSA, 113 controls	Roseburia, Lachnospiraceae, Ruminococcus	Enterobacteriaceae, Akkermansia, Verrucomicrobiaceae, Lactobacillaceae, Oscillospira	Lachnospiraceae (including Roseburia) exhibited a negative association with disease duration, while Lactobacillaceae (including Lactobacillus) and Akkermansia (as well as Verrucomicrobia and Verrucomicrobiaceae) showed a positive association with disease duration. Patients with higher abundance in Christensenellaceae	[13] [2] [3]

			demonstrated more severe non-motor symptoms.	
197 PD patients, 103 controls	family decreased: Lachnospiraceae<br>genus decreased: Roseburia, Faecalibacterium	Family increased: Christensenellaceae, Desulfovibrionaceae;<br>genus increased: Bilophila, Akkermansia;	Parkinson's microbiota was characterized by reduced carbohydrate fermentation and butyrate synthesis capacity and increased proteolytic fermentation and production of deleterious amino acid metabolites, including p-cresol and phenylacetylglutamine. Taxonomic shifts and elevated proteolytic metabolites were strongly associated with stool consistency and constipation among patients.	[14] [3]
147 PD patient, 162 controls	Turicibacter	Anaerotruncus, Christensenella, Lactobacillus, Streptococcus, Akkermansia, Bilophila	The Hoehn and Yahr staging revealed a negative association with Paraprevotella, while showing a positive	[15] [2]

			association with Bilophila	
Training set<br>PD: 40<br>healthy: 40<br>Validation set<br>PD: 78<br>healthy: 75<br>MSA: 40<br>AD: 25		Family increased: Carnobacteriaceae, Lactobacillaceae, Rikenellaceae, Streptococcaceae, Synergistaceae<br>Genus increased: Alistipes, Enterobacter, Gordonibacter, Granulicatella, Holdemania, Lactobacillus, Streptococcus<br>Species increased: Clostridium_asparagiforme, Clostridium_leptum, Enterobacter_cloacae, Gordonibacter_pamelaeae, Granulicatella_unclassified, Holdemania_filiformis, Lachnospiraceae_bacterium 1_1_57FAA, Lachnospiraceae_bacterium 3_1_57FAA_CT1, Lactobacillus_salivarius, Paraprevotella_clara, Streptococcus_anginosus,	Twenty-five gene markers were identified that distinguished Parkinson's disease patients from healthy control subjects, achieving an area under the receiver operating characteristic curve (AUC) of 0.896 (95% confidence interval: 83.1-96.1%).<br>This classifier also performed a differential diagnosis power in discriminating MSA and Alzheimer’s disease.	[16]<br>[3]

		Streptococcus_salivarius, Streptococcus_thermophilus		
26 PD patient, 25 controls	species decreased: Prevotella copri, Clostridium saccharolyticum, Desulfibrio piger	Species increased: Akkermansia muciniphila, Alistipes shahii, Alistipes obesi, Alistipes ihumii;	Functional analysis reveals an increased microbial capability to degrade mucin and host glycans in PD. Personalized community-level metabolic modeling reveals the microbial contribution to folate deficiency and hyperhomocysteinemia observed in patients with PD.	[17] [3]
104 PD patient, 96 controls		Family increased: Christensenellaceae, Verrucomicrobiaceae, Synergistaceae, Catabacteriaceae, Lactobacillaceae; genus increased: Cloacibacillus, Catabacter, Christensenella, Butyrivibrio, Bifidobacterium, Megasphaera; species increased:	low SCFAs in PD were significantly associated with poorer cognition and low BMI. Lower butyrate levels correlated with worse postural instability-gait disorder scores.	[18] [3]

		Bacteroides fragilis, Lactobacillus acidophilus		
490 PD patient, 234 controls			Genus: 23 ↑, 11 ↓; species: 55 ↑, 29 ↓	[19] [3]
96 PD patient, 74 controls		Proteobacteria, Actinobacteria, Enterococcus, Verrucomicrobiota, Akkermansia, Ruminococcaceae, Hungatella	Extended duration of PD was found to be correlated with decreased levels of the Synergistota phylum and significant shifts in six genera. There were increased levels of Fournierella, DTU089, and Haemophilus, as well as decreased levels of Pseudomonas, Lactobacillus, and Roseburia. In addition, two genera were linked to higher UPDRS III scores: an increased level of Lachnospiraceae_ NK4B4_ group and a	[20] [2]

			decreased level of Senegalimassilia.	
48+47 PD patients, 29+30 household controls, 41+49 population controls	Faecalibacterium, Roseburia genera, Faecalibacterium prausnitzii, Eubacterium	Actinobacteria, Bifidobacterium bifidum, Eisenbergiella tayi, Ruthenibacterium lactatiformans, Akkermansia muciniphila	Pathway enrichment analysis reveals disruptions in microbial carbohydrate and lipid metabolism and increased amino acid and nucleotide metabolism in PD. Global gene-level signatures indicate an increased response to oxidative stress, decreased cellular growth and microbial motility, and disrupted intercommunity signaling.	[21] [2]
42 PD patients, 42 controls	Firmicutes, Coriobacteriales Incertae Sedis, Lachnospiraceae ND3007 group, Eubacterium hallii group, Tyzzerella, Ruminococcus	Verrucomicrobiota Lactobacillaceae, Akkermansiaceae, Lactobacillus	disease duration influenced microbiota composition, which in turn influenced development of non-motor symptoms in PD.	[22] [2]

	gauvreauii group, Fusicatenibacter, Prevotella			
Meta-analysis, including 2269 samples by 16S rRNA gene and 236 samples by shotgun metagenomics,	Roseburia, Faecalibacterium, Blautia, Lachnospira, and Prevotella	Streptococcus, Bifidobacterium, Lactobacillus, Akkermansia, and Desulfovibrio	Relative abundances of potential pro-inflammatory bacteria, genes and pathways were significantly increased in PD, while potential anti-inflammatory bacteria, genes and pathways were significantly decreased. lead to a decrease in potential anti-inflammatory substances (short-chain fatty acids) and an increase in potential proinflammatory substances (lipopolysaccharides, hydrogen sulfide and glutamate)	[23]

iRBD, idiopathic REM-sleep behavior disorder; MSA, multiple system atrophy; PD, Parkinson's disease; PSP, progressive supranuclear palsy

References

1. Scheperjans, F.; Aho, V.; Pereira, P. A.; Koskinen, K.; Paulin, L.; Pekkonen, E.; Haapaniemi, E.; Kaakkola, S.; Eerola-Rautio, J.; Pohja, M.; Kinnunen, E.;

Murros, K.; Auvinen, P., Gut microbiota are related to Parkinson's disease and clinical phenotype. *Mov Disord* **2015,** 30, (3), 350-8.

2. Feng, M.; Zou, Z.; Shou, P.; Peng, W.; Liu, M.; Li, X., Gut microbiota and Parkinson's disease: potential links and the role of fecal microbiota transplantation. *Front Aging Neurosci* **2024,** 16, 1479343.
3. Zhang, X.; Tang, B.; Guo, J., Parkinson's disease and gut microbiota: from clinical to mechanistic and therapeutic studies. *Transl Neurodegener* **2023,** 12, (1), 59.
4. Unger, M. M.; Spiegel, J.; Dillmann, K. U.; Grundmann, D.; Philippeit, H.; Burmann, J.; Fassbender, K.; Schwiertz, A.; Schafer, K. H., Short chain fatty acids and gut microbiota differ between patients with Parkinson's disease and age-matched controls. *Parkinsonism Relat Disord* **2016,** 32, 66-72.
5. Petrov, V. A.; Saltykova, I. V.; Zhukova, I. A.; Alifirova, V. M.; Zhukova, N. G.; Dorofeeva, Y. B.; Tyakht, A. V.; Kovarsky, B. A.; Alekseev, D. G.; Kostryukova, E. S.; Mironova, Y. S.; Izhboldina, O. P.; Nikitina, M. A.; Perevozchikova, T. V.; Fait, E. A.; Babenko, V. V.; Vakhitova, M. T.; Govorun, V. M.; Sazonov, A. E., Analysis of Gut Microbiota in Patients with Parkinson's Disease. *Bull Exp Biol Med* **2017,** 162, (6), 734-737.
6. Hill-Burns, E. M.; Debelius, J. W.; Morton, J. T.; Wissemann, W. T.; Lewis, M. R.; Wallen, Z. D.; Peddada, S. D.; Factor, S. A.; Molho, E.; Zabetian, C. P.; Knight, R.; Payami, H., Parkinson's disease and Parkinson's disease medications have distinct signatures of the gut microbiome. *Mov Disord* **2017,** 32, (5), 739-749.
7. Mertsalmi, T. H.; Aho, V. T. E.; Pereira, P. A. B.; Paulin, L.; Pekkonen, E.; Auvinen, P.; Scheperjans, F., More than constipation - bowel symptoms in Parkinson's disease and their connection to gut microbiota. *Eur J Neurol* **2017,** 24, (11), 1375-1383.
8. Bedarf, J. R.; Hildebrand, F.; Coelho, L. P.; Sunagawa, S.; Bahram, M.; Goeser, F.; Bork, P.; Wullner, U., Functional implications of microbial and viral gut metagenome changes in early stage L-DOPA-naive Parkinson's disease patients. *Genome Med* **2017,** 9, (1), 39.
9. Li, W.; Wu, X.; Hu, X.; Wang, T.; Liang, S.; Duan, Y.; Jin, F.; Qin, B., Structural changes of gut microbiota in Parkinson's disease and its correlation with clinical features. *Sci China Life Sci* **2017,** 60, (11), 1223-1233.
10. Heintz-Buschart, A.; Pandey, U.; Wicke, T.; Sixel-Doring, F.; Janzen, A.; Sittig-Wiegand, E.; Trenkwalder, C.; Oertel, W. H.; Mollenhauer, B.; Wilmes, P., The nasal and gut microbiome in Parkinson's disease and idiopathic rapid eye movement sleep behavior disorder. *Mov Disord* **2018,** 33, (1), 88-98.
11. Qian, Y.; Yang, X.; Xu, S.; Wu, C.; Song, Y.; Qin, N.; Chen, S. D.; Xiao, Q.,

Alteration of the fecal microbiota in Chinese patients with Parkinson's disease. *Brain Behav Immun* **2018,** 70, 194-202.

12. Aho, V. T. E.; Pereira, P. A. B.; Voutilainen, S.; Paulin, L.; Pekkonen, E.; Auvinen, P.; Scheperjans, F., Gut microbiota in Parkinson's disease: Temporal stability and relations to disease progression. *EBioMedicine* **2019,** 44, 691-707.
13. Barichella, M.; Severgnini, M.; Cilia, R.; Cassani, E.; Bolliri, C.; Caronni, S.; Ferri, V.; Cancello, R.; Ceccarani, C.; Faierman, S.; Pinelli, G.; De Bellis, G.; Zecca, L.; Cereda, E.; Consolandi, C.; Pezzoli, G., Unraveling gut microbiota in Parkinson's disease and atypical parkinsonism. *Mov Disord* **2019,** 34, (3), 396-405.
14. Cirstea, M. S.; Yu, A. C.; Golz, E.; Sundvick, K.; Kliger, D.; Radisavljevic, N.; Foulger, L. H.; Mackenzie, M.; Huan, T.; Finlay, B. B.; Appel-Cresswell, S., Microbiota Composition and Metabolism Are Associated With Gut Function in Parkinson's Disease. *Mov Disord* **2020,** 35, (7), 1208-1217.
15. Baldini, F.; Hertel, J.; Sandt, E.; Thinnes, C. C.; Neuberger-Castillo, L.; Pavelka, L.; Betsou, F.; Kruger, R.; Thiele, I.; Consortium, N.-P., Parkinson's disease-associated alterations of the gut microbiome predict disease-relevant changes in metabolic functions. *BMC Biol* **2020,** 18, (1), 62.
16. Qian, Y.; Yang, X.; Xu, S.; Huang, P.; Li, B.; Du, J.; He, Y.; Su, B.; Xu, L. M.; Wang, L.; Huang, R.; Chen, S.; Xiao, Q., Gut metagenomics-derived genes as potential biomarkers of Parkinson's disease. *Brain* **2020,** 143, (8), 2474-2489.
17. Rosario, D.; Bidkhori, G.; Lee, S.; Bedarf, J.; Hildebrand, F.; Le Chatelier, E.; Uhlen, M.; Ehrlich, S. D.; Proctor, G.; Wullner, U.; Mardinoglu, A.; Shoaie, S., Systematic analysis of gut microbiome reveals the role of bacterial folate and homocysteine metabolism in Parkinson's disease. *Cell Rep* **2021,** 34, (9), 108807.
18. Tan, A. H.; Chong, C. W.; Lim, S. Y.; Yap, I. K. S.; Teh, C. S. J.; Loke, M. F.; Song, S. L.; Tan, J. Y.; Ang, B. H.; Tan, Y. Q.; Kho, M. T.; Bowman, J.; Mahadeva, S.; Yong, H. S.; Lang, A. E., Gut Microbial Ecosystem in Parkinson Disease: New Clinicobiological Insights from Multi-Omics. *Ann Neurol* **2021,** 89, (3), 546-559.
19. Wallen, Z. D.; Demirkan, A.; Twa, G.; Cohen, G.; Dean, M. N.; Standaert, D. G.; Sampson, T. R.; Payami, H., Metagenomics of Parkinson's disease implicates the gut microbiome in multiple disease mechanisms. *Nat Commun* **2022,** 13, (1), 6958.
20. Zhang, K.; Paul, K. C.; Jacobs, J. P.; Chou, H. L.; Duarte Folle, A.; Del Rosario, I.; Yu, Y.; Bronstein, J. M.; Keener, A. M.; Ritz, B., Parkinson's Disease and the Gut Microbiome in Rural California. *J Parkinsons Dis* **2022,** 12, (8), 2441-2452.

21. Boktor, J. C.; Sharon, G.; Verhagen Metman, L. A.; Hall, D. A.; Engen, P. A.; Zreloff, Z.; Hakim, D. J.; Bostick, J. W.; Ousey, J.; Lange, D.; Humphrey, G.; Ackermann, G.; Carlin, M.; Knight, R.; Keshavarzian, A.; Mazmanian, S. K., Integrated Multi-Cohort Analysis of the Parkinson's Disease Gut Metagenome. *Mov Disord* **2023,** 38, (3), 399-409.
22. Babacan Yildiz, G.; Kayacan, Z. C.; Karacan, I.; Sumbul, B.; Elibol, B.; Gelisin, O.; Akgul, O., Altered gut microbiota in patients with idiopathic Parkinson's disease: an age-sex matched case-control study. *Acta Neurol Belg* **2023,** 123, (3), 999-1009.
23. Nie, S.; Wang, J.; Deng, Y.; Ye, Z.; Ge, Y., Inflammatory microbes and genes as potential biomarkers of Parkinson's disease. *NPJ Biofilms Microbiomes* **2022,** 8, (1), 101.
